# Supplementary material for: The Transcription Factor SCX is a Potential Serum Biomarker of Fibrotic Diseases
Source: Int J Mol Sci. 2020 Jul 16;21(14):5012. doi: 10.3390/ijms21145012 (PMC7404299; doi:10.3390/ijms21145012)

Original blots Figure 8

SCX
Lanes 5-8

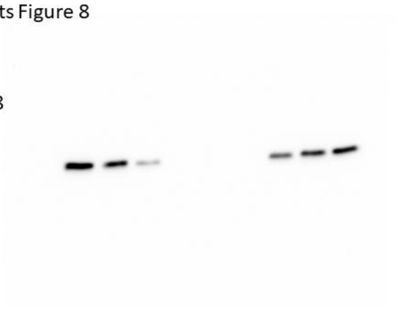

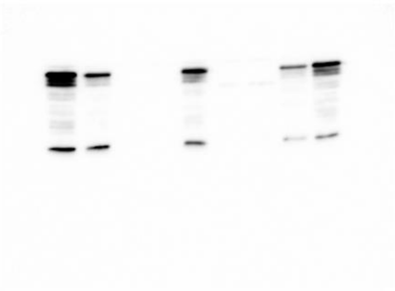

TCF3
Lanes 5-8

SMA
Lanes 5-8

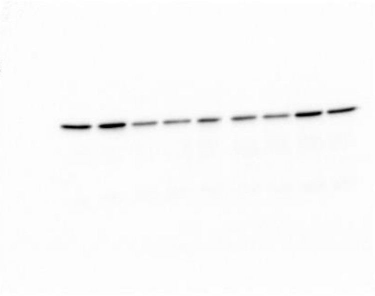

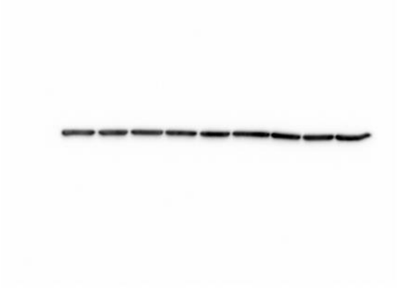

Actin
Lanes 5-8

SCX
Figure 8
MW

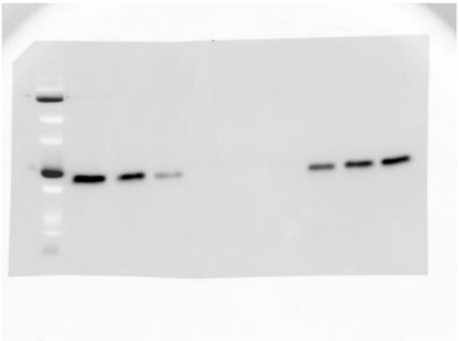

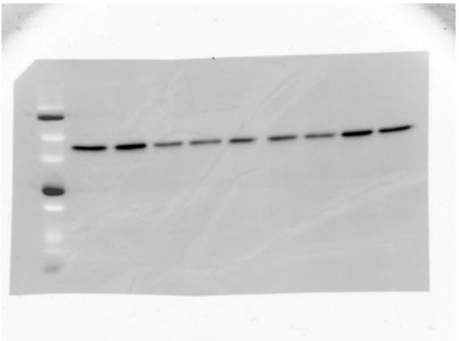

SMA
Figure 8
MW

Actin
Figure 8
MW

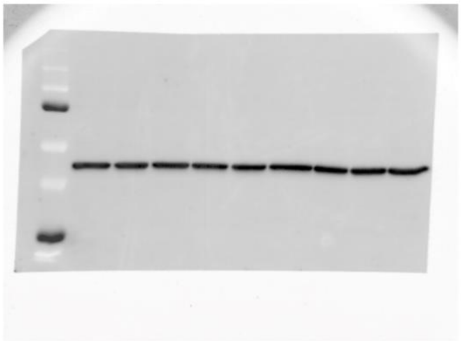

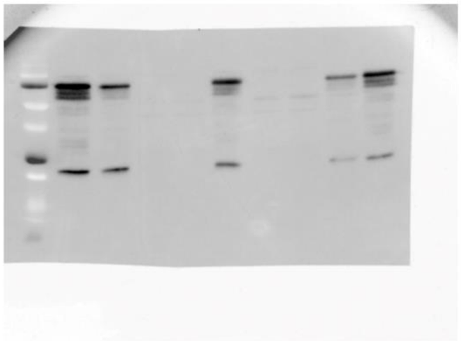

TCF3
Figure 8
MW

Original blots for Supplementary Figures S5 and S6

Lanes 1-5

Lanes 2-9

SCX
Figure S5

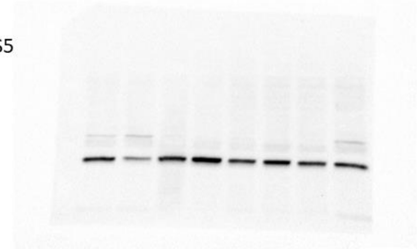

Actin
Figure S5

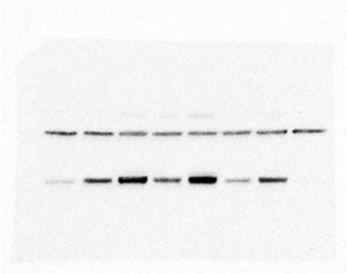

SCX
Figure S6

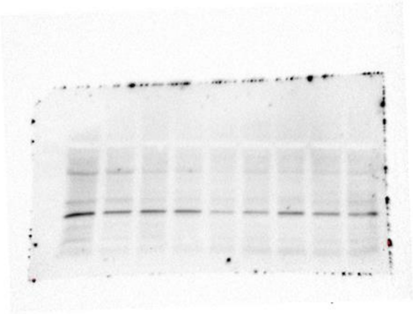

Actin
Figure S6

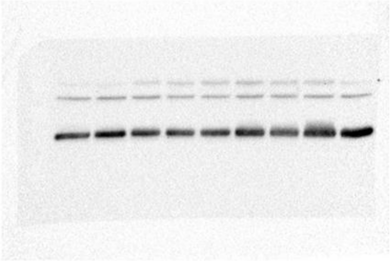

Original blots for Supplementary Figure S7

SCX Lanes 3,4

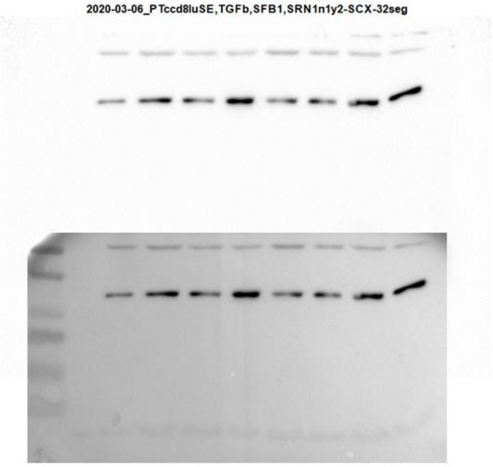

Vinculin Lanes 3,4

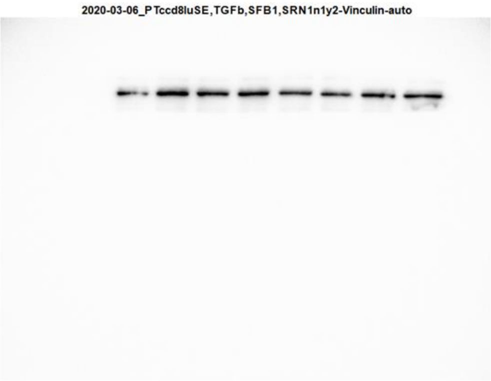

Supplement: Supplementary file 1 [file ijms-21-05012-s001.zip › Original blots .pdf]
