# Supplementary material for: The Transcription Factor SCX is a Potential Serum Biomarker of Fibrotic Diseases
Source: Int J Mol Sci. 2020 Jul 16;21(14):5012. doi: 10.3390/ijms21145012 (PMC7404299; doi:10.3390/ijms21145012)
Supplement: Supplementary file 1 [file ijms-21-05012-s001.zip › Supplementary Figures.docx]

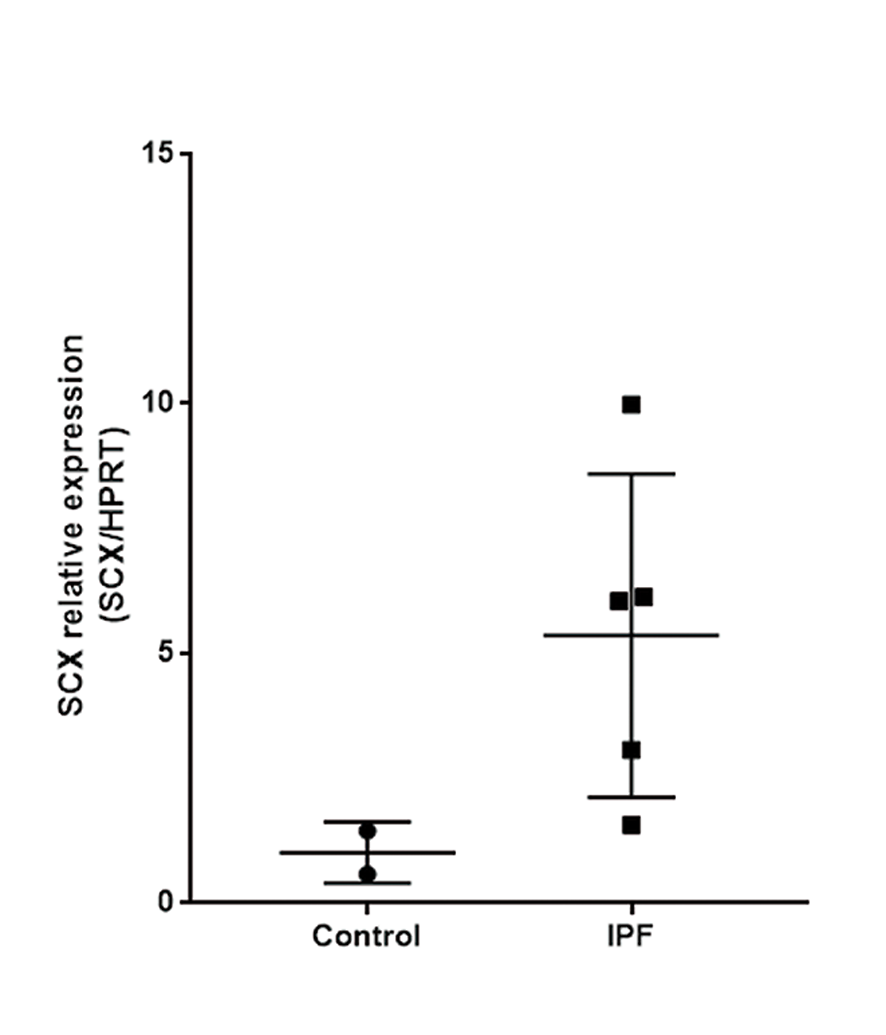


**Figure S1.** SCX gene expression was measured in pulmonary tissue from five IPF patients and two healthy controls (commercial RNA).


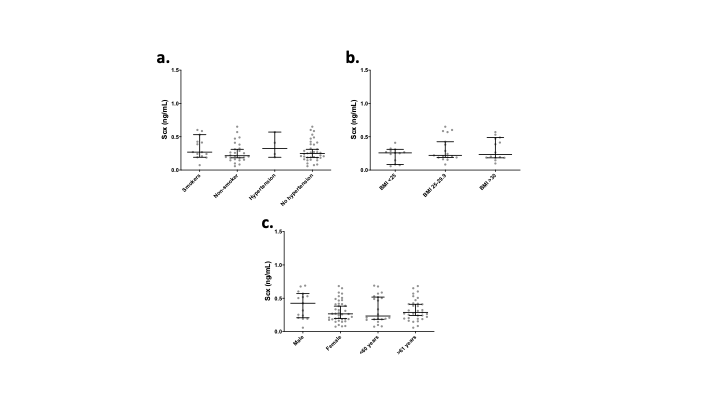


**Figure S2.** No statistical differences in SCX serum levels were found among controls when classified by clinical data. SCX serum levels grouped by clinical characteristics: a) smoking habit and hypertension, b) BMI, and c) sex and age.


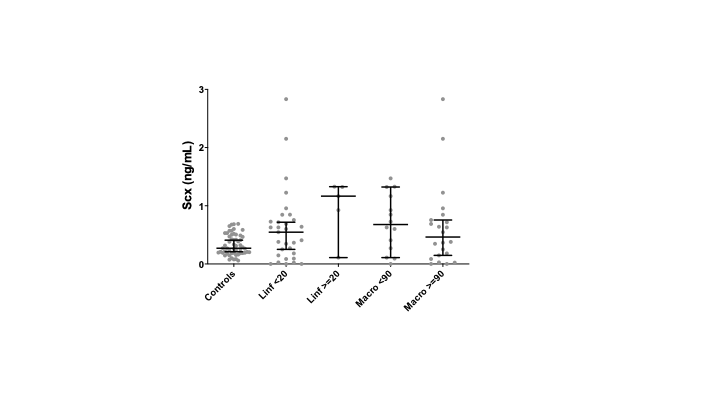


**Figure S3.** No significant differences in SCX serum levels were found when grouped relative to BAL cell populations.


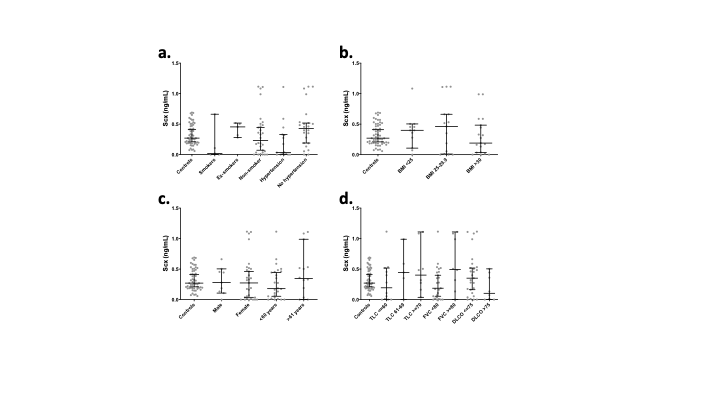


**Figure S4.** SCX serum levels did not differ among clinical groupings of patients with HP. a) smoking habit and hypertension, b) BMI, c) sex and age, and d) spirometry.


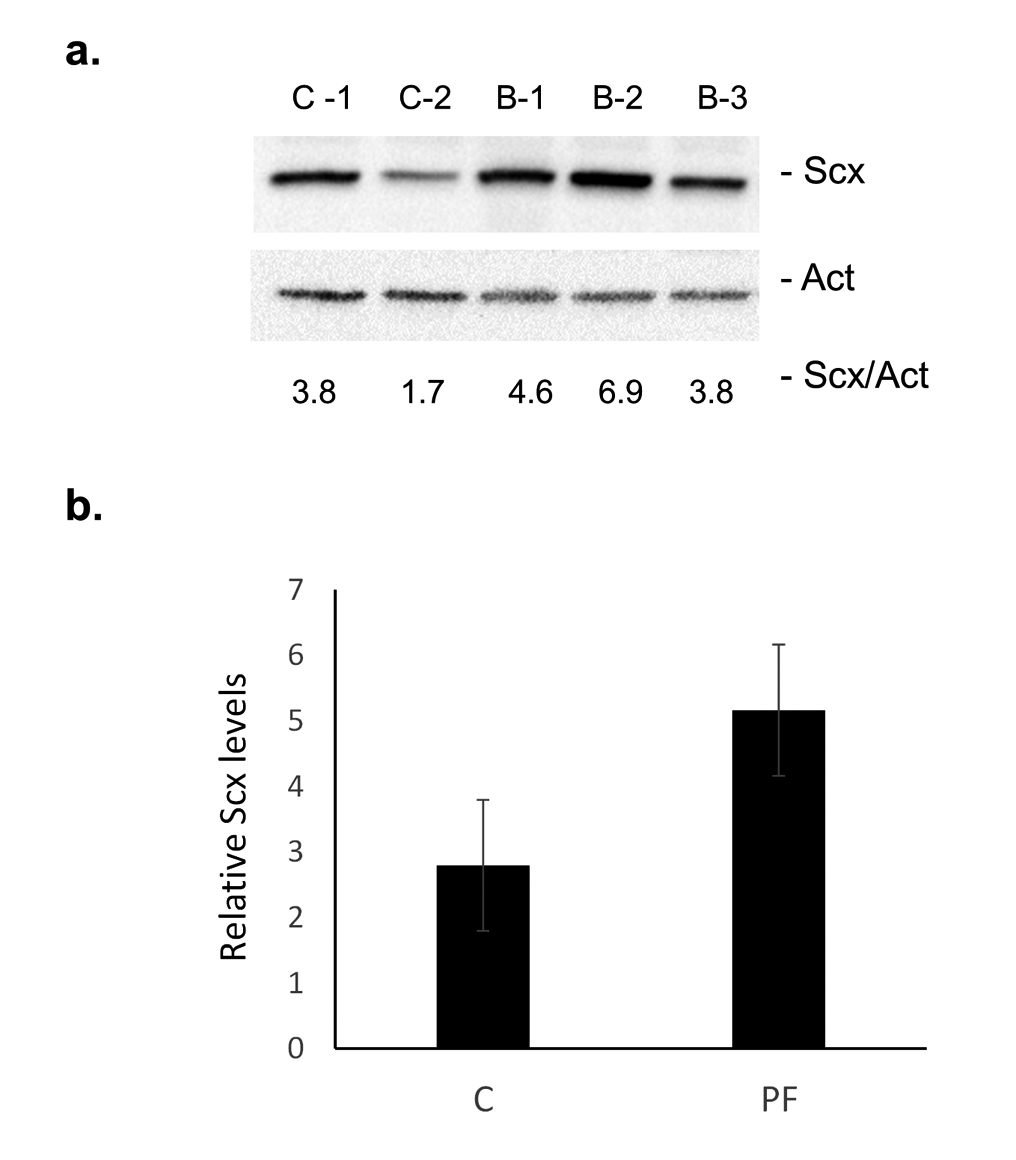


**Figure S5.** SCX protein levels showed a rising trend in mice with bleomycin-induced pulmonary fibrosis. Western blot with lung protein samples derived from control mice (C-1 and C-2) and mice instilled intratracheally with bleomycin to induce pulmonary fibrosis (B-1 to B-3). Mice were sacrificed 28 days after instillation. a) Blot anti-SCX showed variable protein levels, whereas actin levels remained constant. Numbers below blots indicate the relative ratio of densitometries applied to blot bands. b) The graph indicates the average of SCX/Actin ratios for control (C) and pulmonary fibrosis (PF) mice. The presence of pulmonary fibrosis in mice was quantified using the hydroxyproline assay and histology (See Supplementary file 1).


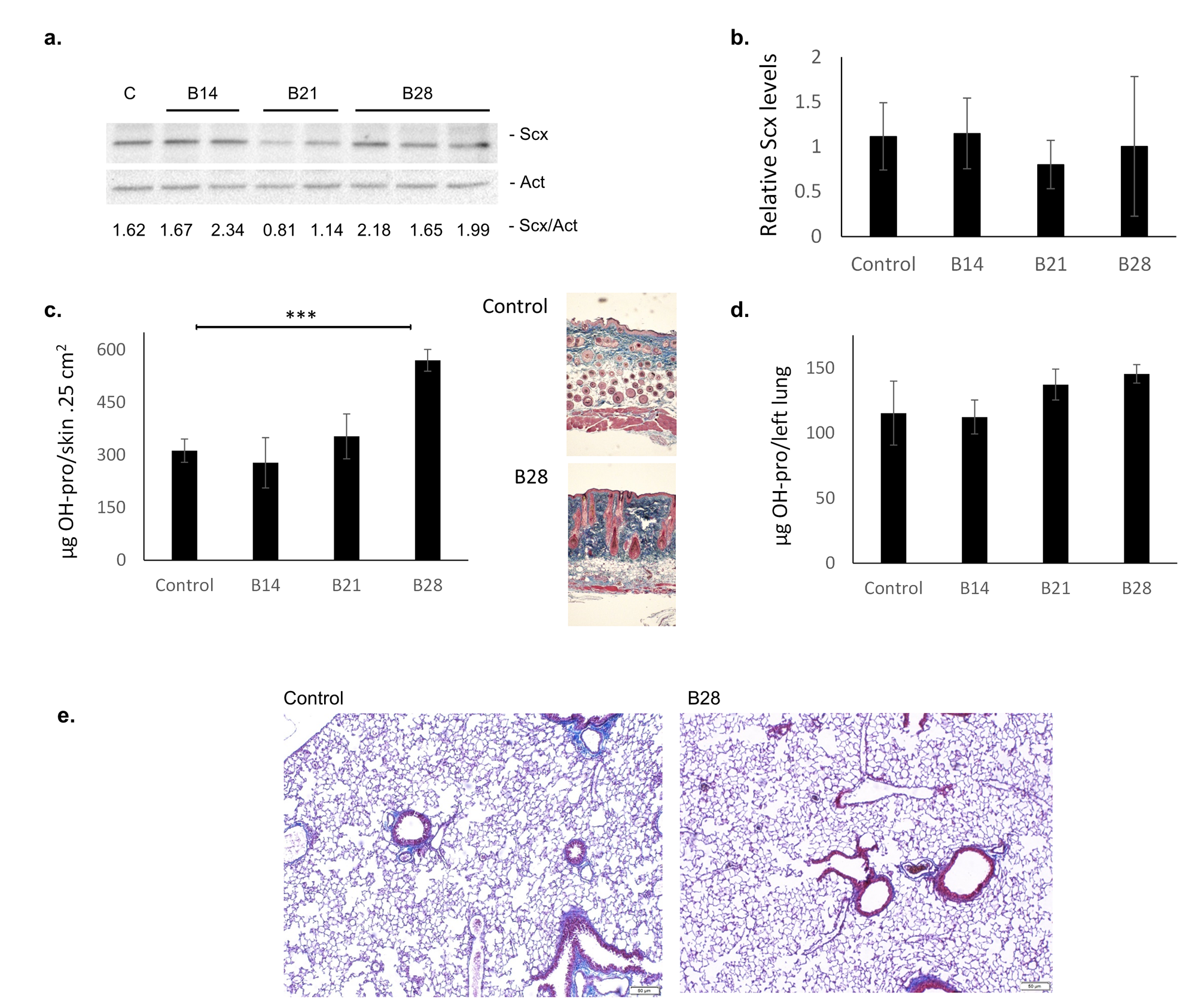


**Figure S6.** Skin SCX protein levels were unaffected in the murine skin fibrosis model. a) Western blot with skin protein samples from control mice (C), and from mice that received subcutaneous bleomycin injections for 14 (B14), 21 (B21) and 28 days (B28) to induce skin fibrosis. Mice were sacrificed one day after the last subcutaneous injection. The blot anti-SCX showed relatively constant protein levels; similarly, actin levels remained constant. Numbers below blots indicate the relative ratio of densitometries applied to blot bands. b) The graph shows the average of SCX/Actin ratios for control (C) and bleomycin-treated mice for the indicated days. c) The presence of skin fibrosis in mice was quantified using the hydroxyproline assay (See Supplementary file 1). Skin treated for 28 days showed statistically higher levels of OH-pro in comparison with all other samples (p<0.001). On the right, two representative images of control and fibrotic skin were included. d) Even though hydroxyproline levels differed slightly between controls and B21 and B28 lung samples, under our experimental conditions, we did not find signs of fibrosis when corresponding lung tissue slices were analyzed with the Masson's trichrome stain (e). Images were taken with the 4X objective of the Olympus microscope IX81, camera DP73.


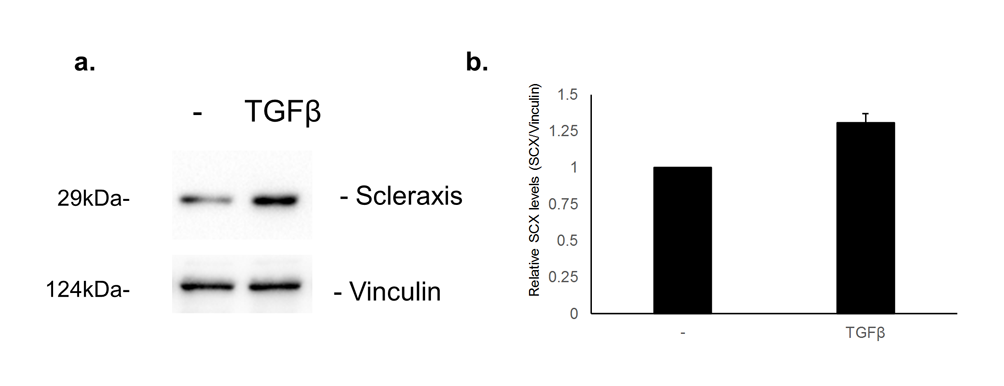


**Figure S7.** TGFβ stimulation in lung fibroblasts promoted a slight increase in SCX expression. (a) Representative immunoblots in CCD8-Lu cells stimulated with 10ng/ml for 24 hours. (b) Densitometric analyses of two independent untreated and TGFβ treated cells. .
