# Supplementary material for: The Transcription Factor SCX is a Potential Serum Biomarker of Fibrotic Diseases
Source: Int J Mol Sci. 2020 Jul 16;21(14):5012. doi: 10.3390/ijms21145012 (PMC7404299; doi:10.3390/ijms21145012)
Supplement: Supplementary file 1 [file ijms-21-05012-s001.zip › Supplementary file 1.docx]

**Supplementary file 1.** Supplementary Methods.

*Bleomycin-induced lung and skin fibrosis*

Animal use complied with international animal care guidelines and was part of the protocol approved by the Bioethics Committee of the Instituto Nacional e Enfermedades Respiratorias “Ismael Cosío Villegas,” Mexico. C57BL/6 background mice were housed under specific pathogen-free facilities and used for experiments between eight and ten weeks of age. All efforts were made to avoid and alleviate animal suffering. Animals received sterile food and water *ad libitum* and were handled aseptically. Bleomycin intra-tracheal instillation was performed as described previously ^1^. Briefly, mice were anesthetized with a mixture of 222-tribromoethanol and t-amyl alcohol by intraperitoneal injection. A single application of 7mg/Kg of bleomycin (Cayman) dissolved in saline solution was surgically instilled in a final volume of 50μl per mouse. Mice were sacrificed 28 days after instillation using pentobarbital overdose. Left lungs were used to measure the content of hydroxyproline, and the right lungs were used for protein extraction.

Skin fibrosis was induced as reported previously ^2^. Briefly, subcutaneous injections of one hundred microliters of 0.5mg/ml bleomycin were performed every other day for four weeks. Injections were made into the shaved back of mice. One day after the final injection, mice were sacrificed by pentobarbital overdose. One cm^2^ of back skin was carefully removed from each animal, of which 0.25 cm^2^ were used for hydroxyproline quantification and 0.5 cm^2^ for protein extraction. Left lungs were also obtained and used for hydroxyproline quantification.

Tissue protein extraction was made with RIPA supplemented with the Set V protease inhibitor cocktail (Calbiochem) and PMSF using the PT6100 polytron. Tissue debris was discarded by high-speed centrifugation, and protein concentration was measured using the Bradford assay.

Collagen quantification was performed using the previously described hydroxyproline assay ^3^, with modifications to measure absorbance using 96 well plates. All tissues were dried for ten days at 110°C. Changes included the use of 5μl of hydrolyzed lung or 1μl of hydrolyzed skin per well. Acid from samples was evaporated from plates, followed by incubations with adjusted volumes of Chloramine T and Ehrlich´s reagent. Hydroxyproline content was calculated using a standard curve, and data were expressed as micrograms of hydroxyproline per lung or per 0.25 cm^2^ of skin.

**References**

1 Valero-Jimenez, A. *et al.* Transmembrane protease, serine 4 (TMPRSS4) is upregulated in IPF lungs and increases the fibrotic response in bleomycin-induced lung injury. *PLoS One* **13**, e0192963, doi:10.1371/journal.pone.0192963 (2018).

2 Yamamoto, T. *et al.* Animal model of sclerotic skin. I: Local injections of bleomycin induce sclerotic skin mimicking scleroderma. *The Journal of investigative dermatology* **112**, 456-462, doi:10.1046/j.1523-1747.1999.00528.x (1999).

3 Woessner, J. F., Jr. & Boucek, R. J. Connective tissue development in subcutaneously implanted polyvinyl sponge. I. Biochemical changes during development. *Archives of biochemistry and biophysics* **93**, 85-94 (1961).
