# Supplementary material for: The Transcription Factor SCX is a Potential Serum Biomarker of Fibrotic Diseases
Source: Int J Mol Sci. 2020 Jul 16;21(14):5012. doi: 10.3390/ijms21145012 (PMC7404299; doi:10.3390/ijms21145012)
Supplement: Supplementary file 1 [file ijms-21-05012-s001.zip › Table S1.docx]

|  | **HIPF231** | **HIPF375** | **HIPF397** | **NOVA** |
| --- | --- | --- | --- | --- |
| **Age** | 64 | 75 | 53 | 30 |
| **Sex (F/M)** | M | F | M | M |

**Table S1.** Characteristics of primary fibroblast donors.
